# Regulation of macroautophagy in ovarian cancer cells in vitro and in vivo by controlling Glucose regulatory protein 78 and AMPK

**DOI:** 10.18632/oncotarget.483

**Published:** 2012-05-06

**Authors:** Prabodh K. Kandala, Sanjay K. Srivastava

**Affiliations:** ^1^ Department of Biomedical Sciences and Cancer Biology Center, Texas Tech University Health Sciences Center, Amarillo, TX, USA

**Keywords:** Diindolylmethane, ER stress, autophagy, apoptosis

## Abstract

In this study we show that diindolylmethane (DIM) induces autophagy in ovarian cancer cells by regulating endoplasmic reticulum (ER) stress and AMPK. Treatment of SKOV-3, OVCAR-3 and TOV-21G ovarian cancer cells with varying concentrations of DIM for 24 hours resulted in a concentration dependent induction of autophagy as measured by flowcytometry. Electron microscopy confirmed the presence of autophagosomes in DIM treated cells. Western blot analysis showed that DIM treatment increased the expression of LC3B, a hall mark of autophagy as well as p62 and Atg 12 proteins that are accumulated during autophagy. Autophagy inhibitors bafilomycin or chloroquine inhibited DIM induced autophagy. Furthermore, DIM treatment significantly increased the expression of ER stress regulators such as Grp78, IRE1 and GADD153. Cycloheximide or ER stress inhibitor mithramycin not only blocked ER stress proteins that were activated by DIM but also autophagy. Silencing Grp78 or GADD 153 significantly blocked the expression of LC3B and p62 indicating that autophagy in our model is mediated by ER stress. Knocking out LC3B inhibited DIM induced autophagy. DIM treatment increased the cytosolic calcium levels which lead to the activation of AMPK in our model. Chelating cytosolic calcium with BAPT-AM abrogated not only the phosphorylation of AMPK but also prevented DIM induced autophagy. Inhibiting AMPK by a chemical inhibitor or siRNA blocked the induction of LC3B or p62, indicating that DIM mediated autophagy requires activation of AMPK. Oral administration of DIM significantly suppressed SKOV-3 tumor xenografts in nude mice. Activation of ER stress and autophagy were observed in the tumors of DIM treated mice. Taken together, these results suggest that induction of autophagy by DIM in ovarian cancer cells was associated with ER stress and AMPK activation.

## INTRODUCTION

Macroautophagy (autophagy hereafter) is a cellular mechanism which involves lysosome-dependent bulk degradation of cytoplasmic components to maintain homeostasis during starvation or stress conditions [1]. It is a highly conserved multi-step process regulated by several ‘Atg’ (Autophagy-related) genes [2]. Autophagic vesicles are formed through nucleation, assembly and elongation of small membrane structures forming double membrane autophagosome. Autophagosome nucleation requires a complex containing Atg6, whereas elongation of autophagosome involves Atg 12 and Atg 8 (LC3 in mammals) [3]. All together, they form autophagic membrane. This membrane assembles around damaged organelles, proteins and cytoplasm, encapsulating the cargo which is degraded during the process of autophagy. Later, the outer membrane of autophagosomes is fused by endosomes or lysosomes to form autolysosomes where lysosomal hydrolases degrade the cytoplasm derived contents of autophagosome together with its inner membrane and presented to citric acid cycle for energy generation [4]. ER stress and activation of AMPK are among the major regulators of autophagy [5].

Endoplasmic Reticulum (ER) or sarcoplasmic reticulum is an important cell organelle that is involved in biosynthesis, protein folding and modification of various soluble and insoluble proteins [6]. ER is also a store house of calcium and maintains its homeostasis [7]. ER is very sensitive to minor perturbations in its environment. Several physiological conditions such as glucose deprivation, oxidative stress and infection can lead to disturbances in the ER called ER stress [8]. ER stress leads to evolutionarily conserved cell stress reponse, the unfolded protein response (UPR) which triggers two downstream signaling events. One is the induction of genes encoding for ER resident chaperones to properly fold the unfolded proteins and the other being suppression of initiation of protein synthesis [9]. These processes are carried out by three transmembrane ER stress sensors, RNA activated protein kinase like ER kinase (PERK), the basic leucine-zipper activating transcription factor ATF6, and the kinase endoribonuclease IRE1α [9]. Several studies suggested that ER stress leads to autophagy [10]. Depending on the context, autophagy counterbalances ER stress induced ER expansion, enhances cell survival or commits the cell to non-apoptotic death. PERK, IRE1 and increased cytosolic calcium have been implicated as mediators of ER stress induced autophagy in mammalian cells [9]. These mediators activate autophagy by upregulating Atg12 and LC3 conversion [11].

ER stress also leads to release of calcium from ER to cytosol, which in turn can activate various kinases that are involved in autophagy signaling [12, 13]. Calcium mediated autophagy is regulated by AMP activated protein kinase (AMPK), which senses cellular energy status to maintain homeostasis. It is usually activated when ATP levels are depleted in the cells. Increase in the cytosolic calcium leads to the activation of Ca^2+^/calmodulin activating kinase kinase β (CAMKKβ) which further activates AMPK [14]. AMPK stimulates autophagy by inhibiting mTORC1 at the level of TSC2 and Raptor. In addition, both AMPK and mTORC1 regulate autophagy through coordinated phoshphorylation of Ulk1 [15, 16].

Ovarian cancer is one of the leading gynecological cancers in United States with high mortality rates. It is usually detected in late stages with poor prognosis [17]. In our previous study, we showed that 3,3’-diindolylmethane (DIM) suppresses the growth of ovarian cancer cells *in vitro* [18]. Here, for the first time we report that DIM activates autophagy by inducing ER stress and phosphorylation of AMPK.

## RESULTS

### DIM induces autophagy in ovarian cancer cells

**Figure 1:**
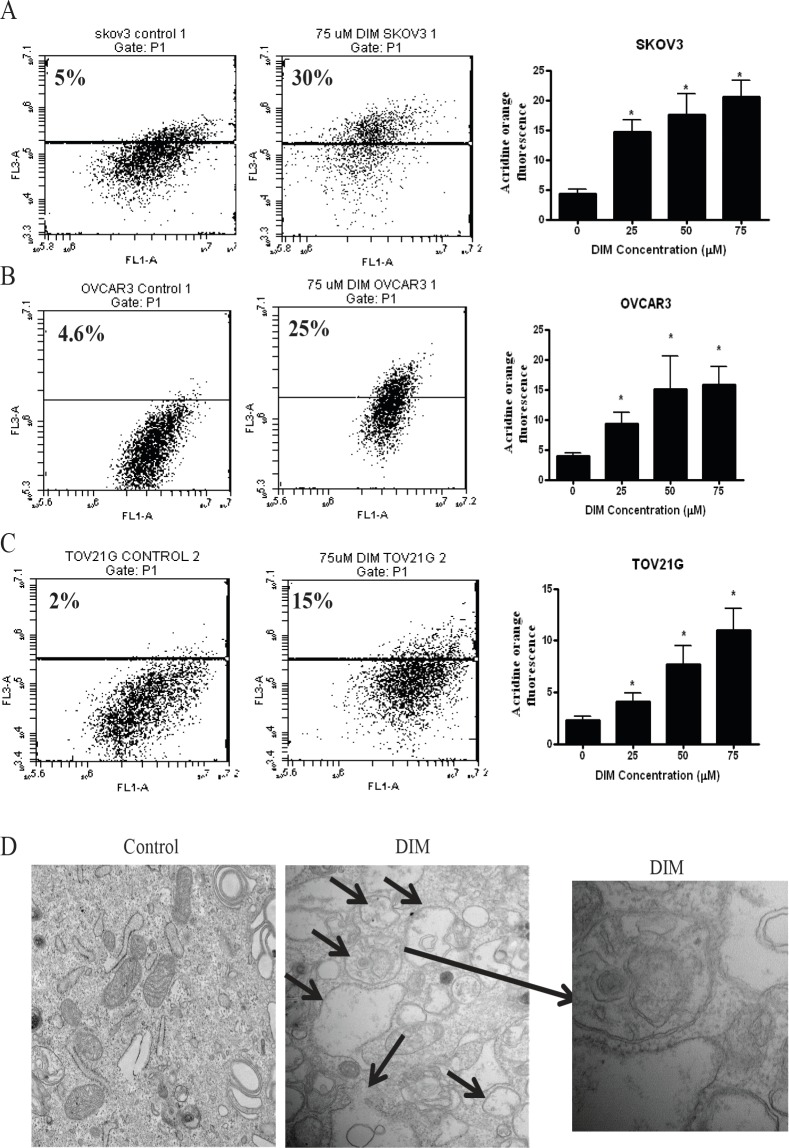
DIM induces autophagy in ovarian cancer cells A) SKOV-3, B) OVCAR-3, C) TOV-21G cells were treated with various concentrations of DIM for 24 hours. Representative dot plots and concentration dependent bar graphs of acridine orange fluorescence are shown. D) Electron microscopy images of control and DIM treated SKOV-3 cells. Means and SD of three independent experiments are shown. Student’s t-test was used for statistical analysis to compare control and DIM treatments. *p<0.05 when compared to control.

Autophagy is activated during stress conditions for degradation and recycling of macromolecules and organelles in the cell. We previously reported that DIM induces cellular stress leading to DNA damage in ovarian cancer cells [18]. Hence, we wanted to determine whether or not DIM induces autophagy in ovarian cancer cells. The autophagy inducing effect of DIM was determined using acridine orange. Acridine orange is a lysomotropic agent that moves freely across biological membranes uncharged. Its protonated form accumulates in acidic compartments during autophagy, where it forms aggregates that fluoresces bright red [19, 20]. Treatment of SKOV-3, OVCAR-3 or TOV-21G cells with various concentrations of DIM for 24 hours resulted in a concentration dependent increase in the number of autophagic cells (Fig 1 A-C). Our results showed that DIM-induced autophagy was nearly 3 to 6 fold in SKOV-3, 2 to 5 fold in OVCAR-3 and 2 to 4 fold in TOV-21G cells, when compared with their respective controls (Fig 1 A-C). For example, 75μM DIM treatment for 24h induced autophagy in approximately 30% in SKOV-3 cells, whereas it was 25% and 15% in OVCAR-3 and TOV-21G cells, respectively (Fig 1A-C). Autophagy induction was further confirmed by electron microscopy. Electron microscopy figures clearly shows autophagosome formation as depicted by accumulation of double membrane vesicles in SKOV3 cells treated with DIM (Fig 1D).

**Figure 2:**
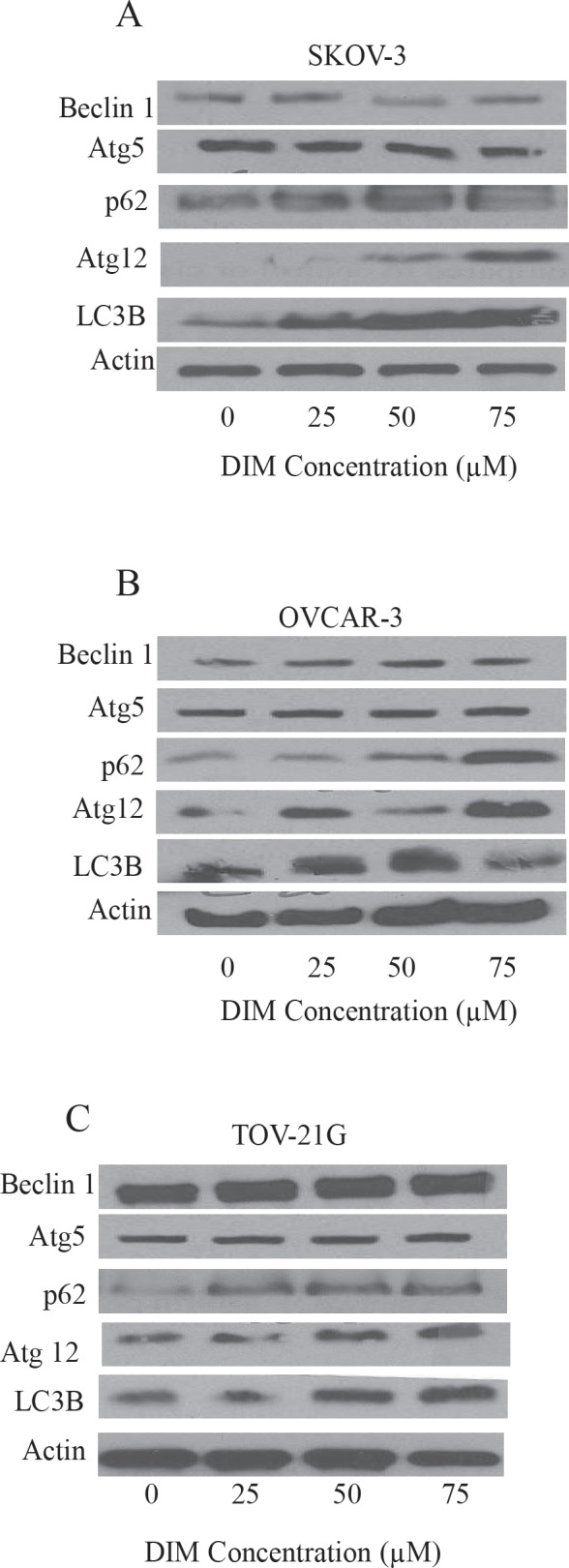
DIM increases the expression of LC3B A) SKOV-3, B) OVCAR-3 and C) TOV-21G cells treated with or without DIM. Representative blots show expression of Beclin1, Atg5, Atg12, P62 and LC3B. Actin was used as loading control

Autophagy inducing effects of DIM were further confirmed by western blot analysis. SKOV-3, OVCAR-3 or TOV-21G cells were exposed to various concentrations of DIM for 24 hours. Our results reveal that DIM upregulates LC3B in a concentration dependent manner in all the cell lines tested (Fig 2 A-C). Our quantitation results showed approximately 2 to 5 fold increase in the expression of LC3B by DIM treatment in different cell lines. DIM induced autophagy was accompanied by increase in the accumulation of Atg12 and p62 (Fig 2 A-C). Autophagy marker p62 is a protein that is selectively incorporated into the autophagosome by directly binding to LC3B and hence aggregate during autophagy [21]. On the other hand, Atg12 is instrumental in the autophagic vesicle biogenesis [3]. DIM treatment failed to exert any effect on Beclin 1 or Atg5 in either of the cell lines tested.

### DIM increases autophagic flux confirming autophagy induction

LC3B is the hallmark of autophagy, however its expression not always means induction of autophagy. Expression of LC3B may represent either the increased generation of autophagosomes or a block in autophagosomal maturation [22]. For example, agents that impair lysosomal acidification such as chloroquine and bafilomycin A1 leads to accumulation of LC3B even under normal conditions because turnover of LC3B by basal autophagy is blocked. Hence, one cannot differentiate between induction of autophagy and impairment of autophagolysosomal maturation simply by measuring levels of LC3B. Hence, it is important to determine autophagic flux using LC3 turnover assay in presence and absence of chloroquine or bafilomycin A1. Ideally, when the cells are treated with lysosomotropic agents such as chloroquine or bafilomycin A1, the degradation of LC3B is blocked, resulting in the accumulation of LC3B [23]. Under these conditions, the difference in LC3B levels in the presence and absence of chloroquine or bafilomycin A1 is more under autophagy induced conditions, indicating that autophagic flux is increased [24].

**Figure 3:**
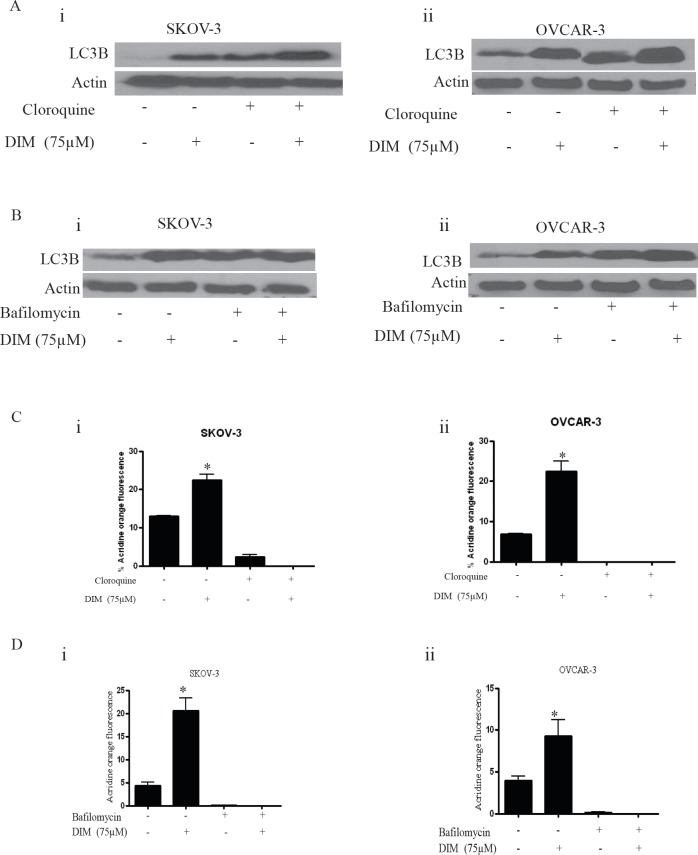
Bafilomycin or chloroquine block DIM induced autophagy in ovarian cancer cells Representative blots showing the expression of LC3B in SKOV-3 or OVCAR-3 cells that were exposed to A) 5μM chloroquine (i-ii) or B) 10 nM bafilomycin (i- ii) for two hours followed by treatment with DIM for 24 hours. Actin was used as loading control. Effect of DIM induced autophagy in presence of C) cloroquine (i, ii) or D) bafilomycin (i, ii) using flowcytometry. The experiments were repeated twice and similar results were obtained. The difference between all the groups was compared by ANOVA. *p<0.05 when compared to control.

Hence, to measure the autophagic flux induced by DIM, SKOV-3 or OVCAR-3 cells were exposed to chloroquine or bafilomycin for one hour followed by treatment with DIM for 24 hours. As expected, bafilomycin or chloroquine treatment alone increased the accumulation of LC3B indicating the blockade of LC3B degradation (Fig 3A-B). The expression of LC3B induced by combination of DIM with bafilomycin or chloroquine was higher compared to the expression of LC3B induced by DIM or lysosomal inhibitor treatments alone indicating that autophagic flux was increased in presence of DIM, yet again confirming that autophagy is induced in our model.

Furthermore, acridine orange assay in presence of chloroquine or bafilomycin was performed to ensure the inhibition of autophagosomal maturation. Since, chloroquine or bafilomycin inhibits lysosomal acidification, acridine orange staining induced by DIM was drastically reduced in presence of these inhibitors (Fig 3C-D). Our results established that bafilomycin or chloroquine treatment blocked DIM induced acridine orange staining in SKOV-3 and OVCAR-3 cells.

### DIM induces ER stress in ovarian cancer cells

**Figure 4:**
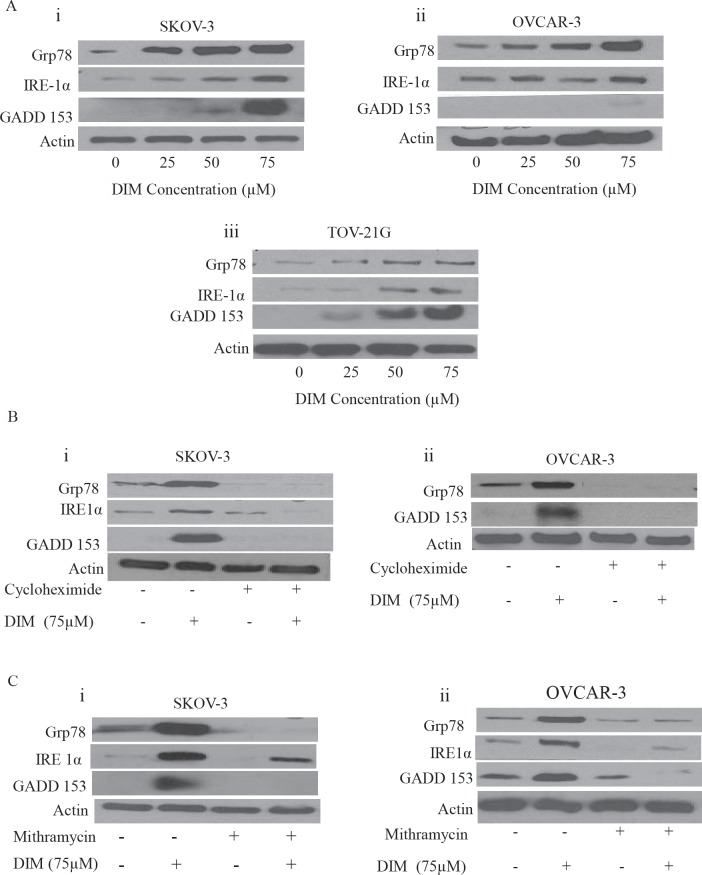
DIM induces ER stress in ovarian cancer cells: A) Representative blots showing the concentration dependent effect of DIM on Grp78, IRE1 and GADD153 in i) SKOV-3, ii) OVCAR-3 and iii) TOV-21G cells B) SKOV-3 (i) or OVCAR-3 (ii) cells were treated with 10μg/ml cycloheximide followed by 75μM DIM for 24 hours. Whole cell lysates were analyzed by western blotting for the expression of Grp78, IRE1 and GADD153. C) Effect of 100nM mithramycin on DIM induced expression of Grp78, IRE1 and GADD153 in i) SKOV-3 or ii) OVCAR-3 is shown. Actin was used as a loading control.

Our next step was to investigate the molecular mechanism behind DIM induced autophagy. Recent literature suggests that disturbances in the integrity of ER activate autophagy [6]. Hence, we questioned ourselves whether DIM causes ER stress in ovarian cancer. To address this issue, SKOV-3, OVCAR-3 or TOV-21G cells treated with varying concentrations of DIM were subjected to western blotting. Our results clearly demonstrate that DIM substantially induces the expression of Grp78 in a concentration dependent manner in all the three cancer cell lines (Fig 4A i-iii). Accumulation of Grp78 is an indicator of ER stress. Our results further show that DIM induced ER stress was accompanied by the accumulation of GADD 153 and IRE1 in SKOV-3, OVCAR-3 and TOV-21G cell lines (Fig 4A i-iii). Both GADD 153 and IRE1 are regulated by Grp78 during ER stress. These observations indicate that DIM induces ER stress in ovarian cancer cells.

### Cycloheximide, a protein synthesis inhibitor or Mithramycin, an ER stress inhibitor block DIM mediated ER stress

Because we observed significantly enhanced protein expression of Grp78, GADD 153 and IRE1 by DIM treatment leading to unfolded protein response (UPR), we wanted to determine whether blocking protein induction would inhibit expression of Grp78, GADD 153 or IRE1. To do that, we used cycloheximide (CHX), a protein synthesis inhibitor. SKOV-3 or OVCAR-3 cells were treated with 10μg/ml CHX for 2 hours followed by treatment with 75μM DIM for 24 hours. Our data reveals that the drastic increase in the expression of Grp78, GADD 153 and IRE1 induced by DIM was attenuated in presence of CHX treatment in SKOV-3 and OVCAR-3 cells (Fig 4B).

CHX is a general protein synthesis inhibitor. To confirm that ER stress is induced in our model, we used mithramycin, a specific ER stress inhibitor. Mithramycin is a gene selective sp1 inhibitor that blocks transcription and protein synthesis of ER chaperones [25].

As shown in Figure 4C, mithramycin treatment blocked the induction of Grp 78, IRE1α and GADD 153. These results strongly support that DIM activates ER stress in ovarian cancer cells.

### Blocking ER stress inhibits DIM induced autophagy

**Figure 5:**
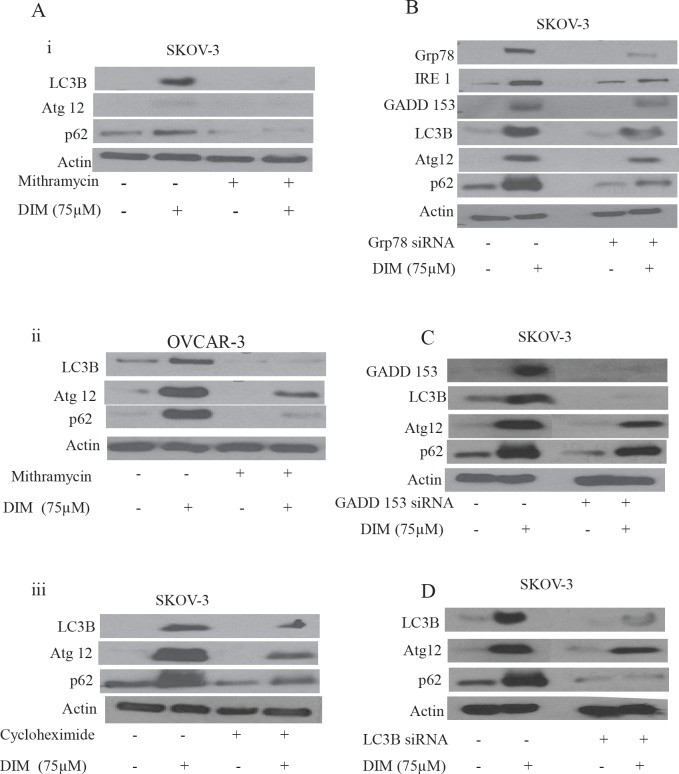
ER stress regulates DIM induced autophagy A) Representative blots showing the expression of Atg12, p62 or LC3B in i) SKOV-3 or ii) OVCAR-3 cells treated with or without DIM in presence of mithramycin. Effect of DIM in presence and absence of CHX in iii) SKOV-3 cells were also represented. Silencing of B) Grp78, C) GADD 153 or D) LC3B using respective siRNA’s in SKOV-3 cells for 48 hours and treated with or without 75 μM DIM for 24 hours. Whole cell lysates were analyzed by western blotting for the expression of Grp78, IRE1, GADD 153, Atg12, P62 or LC3B. Actin was used as a loading control.

Literature suggests that disturbances in ER homeostasis lead to autophagy. Since we observed that ovarian cancer cells exposed to DIM undergo both ER stress and autophagy, we hypothesized that DIM activates autophagy in ovarian cancer cells by inducing ER stress. To establish that ER stress induced by DIM leads to formation of autophagic vesicles, we exposed ovarian cancer cells to mithramycin or CHX before treating cells with DIM. Our results show that mithramycin or CHX treatment not only blocked the enormous increase in the expression of LC3B associated with DIM, but also abrogated the significant upregulation of p62 and Atg12 (Fig 5A i-iii). These results support our hypothesis that ER stress was involved in the regulation of DIM mediated autophagy in ovarian cancer cells.

### Silencing of Grp78, GADD 153 or LC3B inhibits DIM induced autophagy

Both Mithramycin and CHX treatment blocked induction of several molecules that regulate ER stress and autophagy. However, chemical inhibitors are known to be associated with off-target effects. Therefore, to firmly establish the role of ER stress in DIM mediated autophagy, we sought to determine the effects of DIM in SKOV-3 cells in which Grp78 and GADD 153 were transiently silenced using respective siRNA’s. SKOV-3 ovarian cancer cells were transfected with specific siRNA’s followed by treatment with DIM. Fig 5B shows that silencing Grp78 not only abrogated the induction of its downstream molecules IRE1 and GADD 153, but also blocked the DIM induced LC3B, p62 and Atg12. These results indicate that induction of ER stress not only regulates UPR as indicated by expression of IRE1 and GADD 153, but also controls autophagy as evident by the expression of LC3B, p62 and Atg12.

Because silencing Grp78 blocked DIM induced expression of GADD 153, we wanted to evaluate the effect of DIM on SKOV-3 cells after silencing GADD 153. Similar to our Grp78 data, silencing GADD 153 in ovarian cancer cells blocked DIM induced up regulation of LC3B, p62 and Atg12 (Fig 5C). These results confirmed that DIM-induced autophagy was mediated by the activation of ER stress in ovarian cancer cells.

To gain further insight into this mechanism, we silenced LC3B using its specific siRNA. Fig 5D shows that silencing LC3B not only blocked DIM induced expression of LC3B, but also p62 and Atg12 which were associated with autophagy. These results indicate that LC3B was required for the induction of p62 and hence autophagy.

### DIM regulates autophagy by activation of AMPK

**Figure 6:**
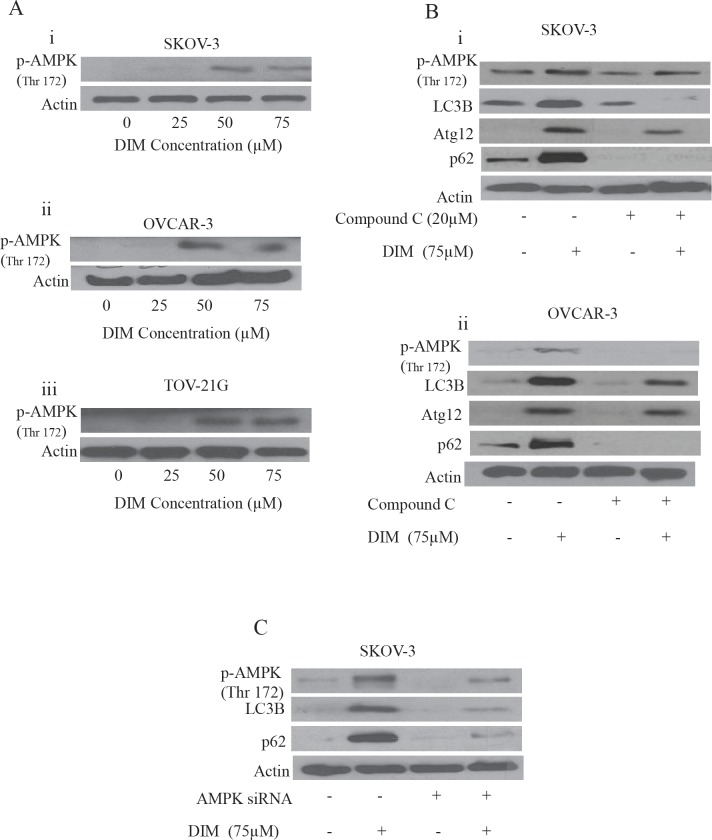
AMPK activation by DIM is necessary for DIM induced autophagy A) Effect of various concentrations of DIM on p-AMPK (Ser 217) in i) SKOV-3, ii) OVCAR-3 or iii) TOV-21G cells. B) Representative blots of p-AMPK, Atg12, P62 or LC3B treated with 75μM DIM in presence or absence of 20μM Compound C in i) SKOV-3 or ii) OVCAR-3 cells. C) Silencing of AMPK using AMPK siRNA in SKOV-3 cells and treated with DIM for 24 hours. Whole cell lysates were analyzed by western blotting for the expression of p-AMPK, P62 and LC3B. Actin was used as a loading control.

Several recent studies showed that activation of AMPK is important in regulating autophagy. We wanted to know whether this was the case in our model. Our western blotting showed that DIM activated AMPK by phosphorylating it at Thr-172 in SKOV-3, OVCAR-3 and TOV-21G ovarian cancer cells (Fig 6A i-iii). To confirm the role of AMPK in DIM induced autophagy, we exposed the cells to compound C, a chemical inhibitor of AMPK before treating with DIM. Our results from Fig 6B shows that treating SKOV-3 or OVCAR-3 cells to compound C inhibited DIM-induced activation of AMPK (Fig6B i-ii). Interestingly, blocking AMPK activation also blocked the expression of LC3B, p62 and Atg 12 in SKOV-3 and OVCAR-3 cells, indicating that AMPK also mediates the effect of DIM on autophagy (Fig 6B i-ii).

To further confirm the role of AMPK, we silenced AMPK by using specific siRNA against AMPK. Genetically silencing AMPK not only inhibited the activation of AMPK by DIM by blocking its phosphorylation at Thr 172, but also abrogated DIM induced expression of LC3B and p62 (Fig 6C). Taken together, these results strongly establish that AMPK is a major regulator of DIM induced autophagy in ovarian cancer cells.

### DIM elevates cytosolic calcium to regulate AMPK and autophagy

**Figure 7:**
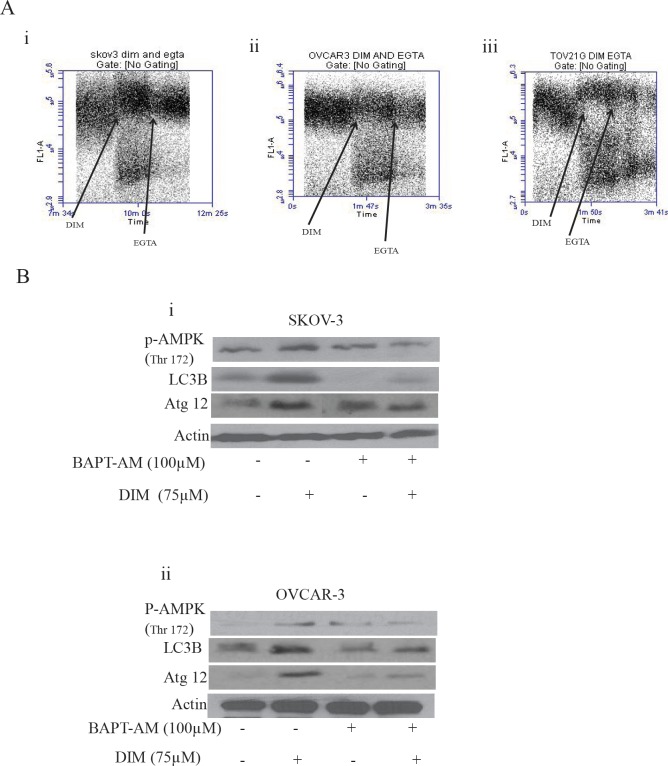
Increase in the cytosolic calcium plays a crucial role in DIM induced autophagy A) SKOV-3 (i), OVCAR-3 (ii) or TOV-21G (iii) cells were loaded with Fluo-3AM dye. Using a flowcytometer, base line reading was taken for a minute. Immediately, cells were exposed to 75 μM DIM for a minute followed by treatment with EGTA for another minute. Increase in the FL-1 intensity is related to increase in the cytosolic calcium levels. B) SKOV-3 (i) or OVCAR-3 (ii) cells were treated with BAPT-AM for one hour followed by treatment with 75μM DIM for 4 hours. Whole cell lysates were analyzed for western blotting for p-AMPK (Ser 217), Atg12 and LC3B. Actin was used as a loading control.

We wanted to gain further insight into how DIM caused activation of AMPK. Many studies suggested that increase in the cytosolic calcium leads to activation of AMPK. It is well known that endoplasmic reticulum is the store house of calcium. Perturbation in the ER results in the release of calcium into cytosol thereby causing deregulation of calcium homeostasis leading to activation of AMPK and autophagy. To test whether DIM treatment leads to the release of calcium in ovarian cancer cells, we loaded cells with Fluo-3AM and continuously monitored calcium levels using Accuri C6 flow cytometer. Baseline reading was taken for a minute, followed by exposure of cells to DIM. Figure 7A clearly demonstrates an increase in the intensity of the baseline after DIM treatment suggesting the release of calcium in different ovarian cancer cells.

To test whether increase in the cytosolic calcium leads to activation of AMPK, we treated SKOV-3 or OVCAR-3 cells with BAPT-AM, a calcium chelater followed by exposure to DIM for 4 hours. We observed that BAPT-AM significantly blocked the activation of AMPK (Fig 7B i-ii). Furthermore, the expression of LC3B or Atg12 induced by DIM was also significantly suppressed by BAPT-AM treatment (Fig 7B i-ii). Taken together, these results suggest that increase in the cytosolic calcium regulates autophagy in DIM treated ovarian cancer cells.

### Tumor growth suppression by DIM is associated with ER stress and autophagy

**Figure 8:**
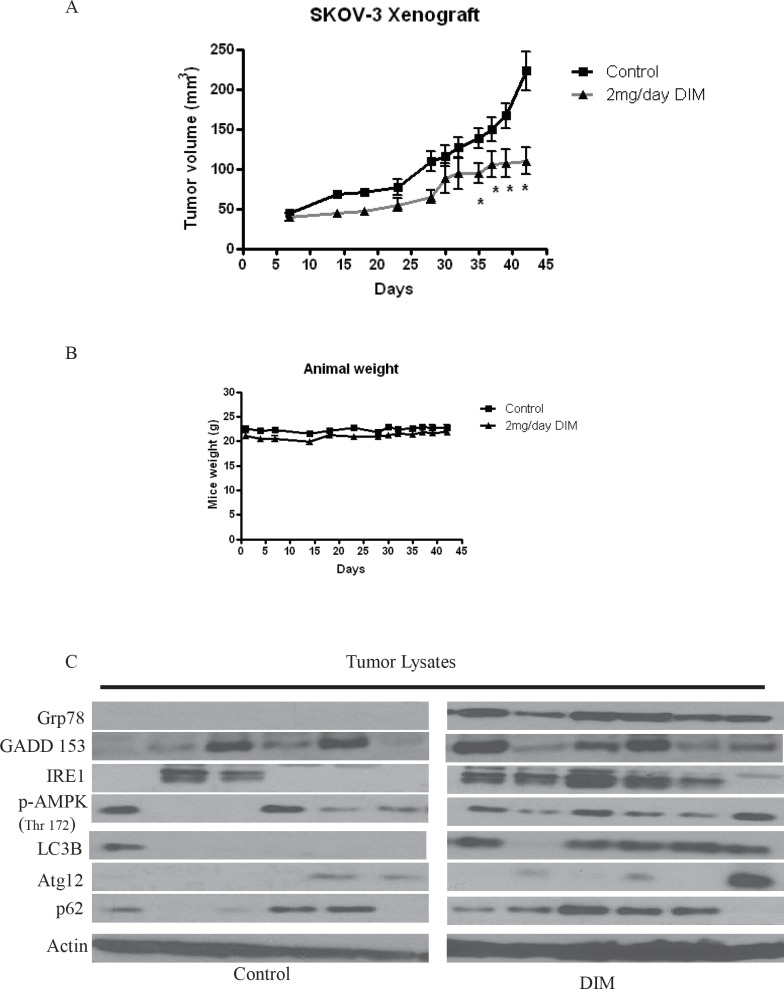
DIM suppresses the growth of ovarian tumors in nude mice by inducing ER stress modulated autophagy SKOV-3 tumor cells were implanted into athymic nude mice. Mice received 2mg/day DIM by oral gavage every day. A) Effect of DIM on ovarian tumor growth. *p<0.05 when compared to control. B) Animal weights during the course of the experiment. C) Expression of ER stress or autophagy molecules in the tumors of mice administered with DIM. Tumors from control and treated mice were excised on day 42 after implantation. Lysates from different tumors were loaded in different lanes and analyzed by western blotting for Grp78, IRE1, GADD 153, Atg12, P62 and LC3B. Blots were stripped and reprobed with actin antibody to verify equal protein loading.

We previously demonstrated that DIM inhibits the growth of various ovarian cancer cells *in vitro* [18]. Whether or not, DIM suppresses the growth of ovarian tumors in vivo was not clear. Hence, we used ovarian tumor xenograft model to determine the growth suppressive effects of DIM in vivo. We injected 5 x 10^6^ SKOV-3 cells subcutaneously on both right and left flanks of female athymic nude mice. Mice were randomized into two groups of 10 mice each. Treated group received 2mg/day DIM by oral gavage, whereas, control group received PBS. Tumor volume was recorded thrice a week using vernier calipers and weight of mice was recorded twice a week. Our results show that DIM significantly suppressed the growth of ovarian tumors. For example, at day 42, average tumor volume in the group of mice that received DIM was approximately 100 mm^3^ which was remarkably lesser as compared to the average tumor volume of 250 mm^3^ in control group (Fig 8A). Interestingly, weight of mice from both groups did not differ significantly suggesting that DIM was not toxic to the mice (Fig 8B). To test whether DIM mediated tumor growth suppression was associated with ER stress and autophagy, tumors from control and DIM treated mice were lysed and subjected to western blotting. As expected, our results show that DIM activates ER stress and autophagy in vivo. We observed an increase in several ER stress markers such as Grp78, GADD 153 and IRE1 in the tumors of DIM treated mice as compared to controls (Fig 8C). Moreover, we observed an increase in the phosphorylation of AMPK and expression of other autophagy markers such as LC3B and p62 in DIM treated tumors (Fig 8C). These results suggest that DIM suppressed the growth of ovarian tumors in vivo by inducing ER stress mediated autophagy.

### DISCUSSION

Our study for the first time demonstrates that DIM treatment induces autophagy in ovarian tumor cells *in vitro* and *in vivo* by enhancing ER stress and activating AMPK. Autophagy is a catabolic process during which damaged organelles and proteins are engulfed and degraded to provide metabolic needs. Autophagy is activated in response to various kinds of stress. Our published studies showed that DIM induces cellular stress in ovarian cancer cells [18]. Our current results suggest that DIM induces autophagy in SKOV-3, OVCAR-3 and TOV-21G human ovarian cancer cells in a concentration dependent manner as analyzed by flowcytometry and electron microscopy.

LC3B is accumulated and localized to vesicular structures during autophagy and hence it is considered as a hallmark of autophagy. Activation of autophagy by DIM in our model was confirmed by enhanced expression of LC3B. Our results also showed that autophagy induction was associated with an increase in the expression of p62 and Atg12. Autophagy marker p62 is a protein that is selectively incorporated into autophagosome by directly binding to LC3B and hence aggregate during autophagy. Atg12 is instrumental in the autophagic vesicle biogenesis [3]. Our studies are in agreement with previous studies, which show that agents such as resveratrol induce autophagy by increasing the expression of LC3B [21]. Beclin 1 and Atg5 are genes that are responsible for initiation of autophagy. However, to our surprise, Beclin1 or Atg5 expression by DIM treatment was not significantly altered. It is interesting to note that several studies have reported that induction of autophagy can be independent of Beclin 1 or Atg5 [26, 27].

DIM treatment in presence of bafilomycin or chloroquine lead to increased expression of LC3B when compared to the cells treated either with DIM or inhibitors alone, suggesting increased autophagic flux. Both bafilomycin and chloroquine inhibit the fusion between autophagosomes and lysosomes, thus prevent the execution step of autophagy. Nonetheless, our results from flowcytometry demonstrated that chloroquine or bafilomycin completely blocked DIM-induced autophagy. Induction of autophagy in ovarian cancer cells was confirmed by genetic silencing of LC3B. Knocking out LC3B inhibited DIM induced expression of LC3B as well as Atg12 and p62. Our observations are in agreement with several studies demonstrating the role of LC3B and p62 in autophagy [21, 28].

Our study established that autophagy induced by DIM treatment was mediated by activation of ER stress or AMPK activation. Activation of ER stress in our model was associated with an increase in the expression of Grp78, IRE1 and GADD153. Grp78 is a chaperone that folds protein into its proper confirmation. Grp78 acts as a sensor in ER and it binds to IRE1 under normal conditions [9]. However, during ER stress, it releases IRE1, which further leads to expression of a transcriptional factor GADD 153 that continues the response of unfolded proteins [5, 9]. Both IRE1 and GADD 153 were significantly up regulated by DIM treatment in all the three ovarian cancer cells. Genetically silencing Grp78 attenuated DIM induced expression of IRE1 and GADD 153, suggesting the importance of Grp78 in regulating DIM induced ER stress.

Furthermore, role of ER stress by DIM induced autophagy was established in our model. Pharmacologically inhibiting ER stress using chemical inhibitors such as cycloheximide or mithramycin not only blocked the induction of Grp78, IRE1 or GADD 153, but also inhibited DIM induced expression of Atg12, LC3B and p62 in SKOV-3 and OVCAR-3 ovarian cancer cells. ER stress is connected to autophagy in our model by IRE-1 and GADD153. IRE1 activates transcriptional factors such as Hac1 which is capable of inducing several Atg genes leading to the induction of autophagy [5, 11, 29]. Accordingly, our results showed that blocking the expression of IRE1 inhibited DIM induced autophagy. During ER stress, Grp78 releases IRE1 leading to the expression of GADD 153. Several studies have demonstrated the involvement of GADD 153 in autophagy [30]. Knocking out Grp78 or GADD 153 blocked the expression of LC3B, Atg12 and p62 induced by DIM. Interestingly knock down of Grp78 also abrogated DIM induced expression of IRE1 and GADD 153, confirming that IRE-1 and GADD 153 are major players of ER stress and regulate autophagy in our model. Similar to our observations, ER stress induced autophagy was observed by capsaicin and atorvastatin in other malignancies [31, 32].

ER stress not only activates UPR, but also leads to the release of calcium from ER into cytosol. Our studies demonstrated that DIM treatment elevates cytosolic calcium levels in ovarian cancer cells. Cytosolic calcium levels were reduced in the presence of EGTA, a calcium chelater. Release of calcium into cytosol leads to the activation of various kinases including AMPK which is known to regulate autophagy. AMPK is also an energy sensor and is activated when there is increase in AMP/ATP ratio, which is usually the scenario during cellular stress, the same reason for which autophagy is activated. In agreement with these facts, DIM treatment activated AMPK in all the three ovarian cancer cell lines. BAPT-AM, a calcium chelater not only suppressed DIM induced phosphorylation of AMPK but also attenuated the expression of LC3B and Atg12. Role of AMPK in DIM induced autophagy was established by silencing AMPK. These studies showed that absence of AMPK inhibits the expression of p62 or LC3B, the classic markers of autophagy, hence confirming that AMPK regulates autophagy in our model. All these observations are in agreement with several studies showing that activation of AMPK leads to autophagy [16, 32].

Oral administration of 2mg DIM everyday substantially suppressed the growth of ovarian tumors. The tumors from DIM treated mice clearly demonstrated the significant induction of ER stress and autophagy similar to our *in vitro* observations. Interestingly, mice that received DIM did not show any significant change in the body weight when compared with the weight of control mice. DIM is an indole compound present in cruciferous vegetables [33]. Recent clinical trials suggested that DIM is well tolerated in humans [34, 35]. Interestingly, administration of 2mg/kg/day DIM through oral route suppressed Cervical Intraepithelial Neoplasia with 1-2 grades [34]. Several pharmacokinetic studies on DIM stated that up to 300mg single dose of DIM can be well tolerated in humans [35]. Our dose of DIM falls within the accepted and tolerated dose in humans. Nevertheless, further clinical studies are needed to show that DIM can reduce the ovarian tumor growth in humans.

In conclusion, DIM promotes autophagy in ovarian cancer through activation of ER stress and AMPK. To our knowledge, this is the first report showing the regulation of autophagy by DIM in ovarian cancer cells.

## MATERIALS AND METHODS

### Chemicals

BR-DIM was a kind gift from Dr. Michael Zeligs (Bio Response, Boulder, CO). Antibodies against Atg5, Beclin, LC3B, Atg12, GADD 153, Grp78, IRE-1α, p-AMPK including AMPK and LC3B siRNA were obtained from Cell Signaling Technology (Danvers, MA). Antibody against p62 was obtained from Abcam Inc (Cambridge, MA). Actin antibody, bafilomycin, cycloheximide, chloroquine, mithramycin, compound C, EGTA, acridine orange and Medium 199 were procured from Sigma Aldrich (St. Louis, MO). RPMI and McCOY 5A were purchased from Mediatech (Manassas, VA). Grp78 siRNA was from Santa Cruz (Santa Cruz, CA). GADD 153 siRNA was from Thermo Scientific (Lafayette, CO). Fluo-3AM was from Invitrogen (Carlsbad, CA) and siPORT neoFX transfection reagent from Applied Biosystems (Foster city, CA)

### Cell culture

SKOV-3, OVCAR-3 and TOV-21G cells were procured from American Type Culture Collection (ATCC, Manassas, VA) and maintained as described previously [18].

### Western blotting

SKOV-3, OVCAR-3 and TOV-21G cells were exposed to varying concentrations of DIM for 24 hours. Cells were collected, lysed and 20-80 μg protein was subjected to SDS gel electrophoresis followed by immunoblotting as previously described by us [18, 36].

### Acridine orange assay

SKOV-3, OVCAR-3 or TOV-21G cells were plated at a density of 0.3 x 10^6^ cells per well in a six-well plate and allowed to attach overnight. Cells were then treated with or without DIM. After 24 hours, cells were washed, suspended in binding buffer and incubated for 15 minutes with 0.1μg/ml acridine orange. Fluorescence was measured using C6 Accuri flowcytometer (Ann Arbor, MI) with a minimum of 10,000 events per sample. In another experiment, cells were treated with 10nM bafilomycin or 5μM chloroquine prior to the treatment with DIM and processed as explained above.

### Transmission electron microscopy

SKOV-3 cells treated with or without DIM were harvested by trypsinization and fixed in ice cold 3% glutaraldehyde overnight.

After washing in PBS, cells were post fixed in Oso4 and embedded in EPON resin. Embedded samples were further processed for ultra-structural analysis as described previously [37].

### Inhibitors treatment

About 0.3 x 10^6^ SKOV-3, OVCAR-3 or TOV-21G cells were treated with either 10nM bafilomycin A1, 100nM mithramycin, 10μg/ml cycloheximide, 5μM chloroquine or 5mg/ml compound c for 2 hours before treating with 75μM DIM for 24 hours. Only in the case where cells were exposed to 10μM BAPT-AM, cells were treated with DIM for 4 hours. After the treatment time, cells were collected and processed for western blotting as explained above.

### Calcium measurement using flowcytometry

Calcium was measured using flowcytometer as described previously [38] with some modifications. About 1 x 10^6^ cells were plated in a 100mm tissue culture dish and allowed to attach in the incubator overnight. Cells were then incubated with Fluo-3AM Dulbecco’s PBS without Ca^2+^ and Mg^2+^ at 37°C for 15 minutes. A base line reading was taken by flowcytometer before addition of DIM. After treating cells with DIM for one minute, EGTA that chelates calcium was used as a negative control.

### In vivo tumor xenograft

Four to six weeks old female athymic nude mice were purchased from Charles River Laboratories (Wilmington, MA). The use of mice and their treatment was approved by Institutional Animal Care and Use Committee (IACUC), Texas Tech University Health Sciences Center. All experiments were carried out in strict compliance with regulations. Mice were fed with an antioxidant-free AIN-76A special diet for a week before starting the experiment. About 5 x 10^6^ SKOV-3 cells were injected subcutaneously into both right and left flanks. Ten mice were assigned randomly to each group. Since each mouse was implanted with two xenografts, each group had twenty tumors. Two days after tumor implantation, mice in the control group received PBS whereas mice in the treatment group received 2mg DIM suspended in PBS by oral gavage every day. Beginning the 7^th^ day after cell implantation, tumor volume was measured thrice a week using vernier calipers until day 42 as previously described by us [36, 39]. At day 42, mice were euthanized and tumors were removed and snap frozen for western blot analysis.

### Statistical Analysis

All the statistical analysis was performed using Prism 5.0 (GraphPad Software Inc., San Diego, CA). The data represents mean values with SEM. Student’s *t*-test were used to compare the control and treated groups. In experiments involving more than three groups, non-parametric analysis of variance followed by Bonferroni post hoc multiple comparision test was used. All statistical tests were two sided. Differences were considered statistically significant when the p value was less than 0.05.
